# Correction to “Natural haplotype variation in *OsGSK3* fine‐tunes grain length via phosphorylating and stabilizing OsRanBP1 in rice”

**DOI:** 10.1111/tpj.71126

**Published:** 2026-09-29

Wei, X. , Du, H. , Yin, J. , Zhan, X. , Zhang, C. , Zhang, J. et al. (2026) Natural haplotype variation in *OsGSK3* fine‐tunes grain length via phosphorylating and stabilizing OsRanBP1 in rice. The Plant Journal, 127, e71079. Available from: 10.1111/tpj.71079.PMC1345863742576696

In Figure 6 panel B and panel E were incorrect. For Figure 6 panel B, in the actual experiment, lanes 1 and 2 correspond to the phosphorylation reaction of GST‐OsGSK3^Hap3^ + MBP‐OsRanBP1, and lanes 6 and 7 correspond to the reaction of GST + MBP‐OsRanBP1. For Figure 6 panel E, the right side of Fig. 6E should be myc‐OsRanBP1 and OsGSK3‐GFP, and it was originally mislabeled as MBP‐OsRanBP1 and GST‐OsGSK3. The correct Figure 6 is provided below:

**Figure 6:**
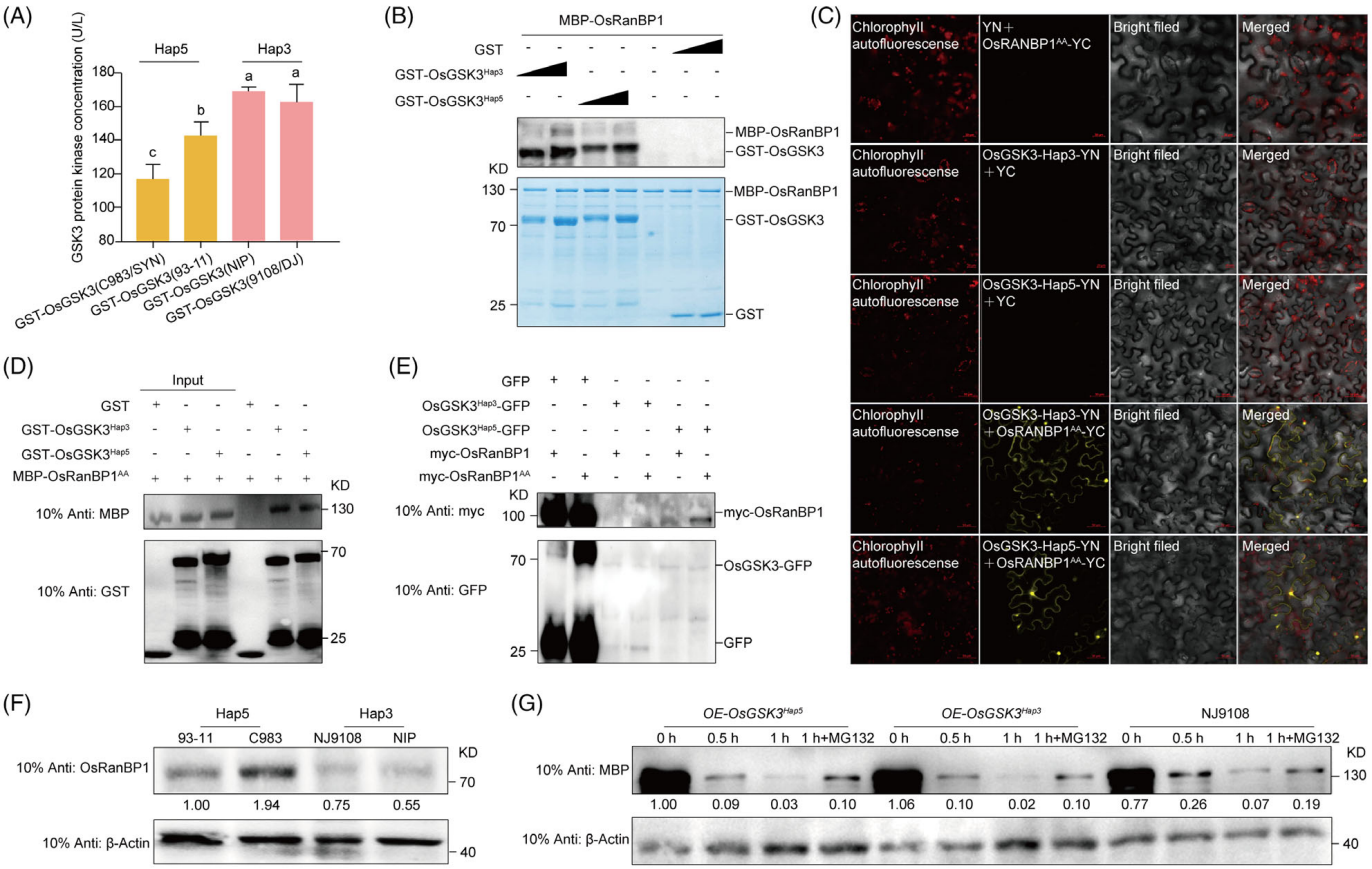
Differential phosphorylation of OsRanBP1 by two OsGSK3 haplotypes. (A) Kinase activity assay of OsGSK3 in six rice varieties: C983/SYN (Hap5), 93‐11 (Hap5), NIP (Hap3), and NJ9108/DJ (Hap3), *n* = 3. Different letters (a–c) indicate significant differences among groups based on one‐way ANOVA followed by Duncan's multiple range test (*P* < 0.05). (B) *In vitro* phosphorylation of OsRanBP1 by OsGSK3^Hap3^ and OsGSK3^Hap5^. Upper panel: Western Blot result; lower panel: Coomassie Brilliant Blue staining result. +: present; −: absent. Triangles indicate increasing protein concentration, *n* = 3. (C) Bimolecular fluorescence complementation (BiFC) assay validating the interactions of OsGSK3^Hap3^ and OsGSK3^Hap5^ with OsRanBP1^AA^, respectively. OsRanBP1^AA^: two phosphorylation sites of OsRanBP1 targeted by OsGSK3 were mutated to alanine. Chlorophyll autofluorescence: chloroplast autofluorescence signal; Bright field: bright‐field image; Merged: merged image. Scale bars are indicated by red lines in the bottom right corner of each panel (20/50 μm). (D) GST pull‐down assay confirming the interactions of OsGSK3^Hap3^ and OsGSK3^Hap5^ with OsRanBP1^AA^. OsRanBP1^AA^: two phosphorylation sites of OsRanBP1 targeted by OsGSK3 were mutated to alanine. +: present; −: absent, *n* = 3. (E) Co‐expression of OsGSK3^Hap3^ and OsGSK3^Hap5^ (with different tags) and OsRanBP1 (wild‐type or mutant) in tobacco leaves. Protein expression levels were detected by western blot after protein extraction. OsRanBP1^AA^: two phosphorylation sites of OsRanBP1 targeted by OsGSK3 were mutated to alanine. +: present; −: absent. (F) Western blot analysis of OsRanBP1 protein expression levels in 93‐11, C983, NJ9108, and NIP, with β‐Actin used as a loading control, *n* = 3. (G) Cell‐free assay detecting the stability of MBP‐OsRanBP1 in the NJ9108, *OE‐OsGSK3* ^*Hap3*^, and *OE‐OsGSK3* ^*Hap5*^ backgrounds. Samples were collected at 0, 0.5, 1, and 1 h after MG132 treatment, with β‐Actin used as a loading control, *n* = 3.

We apologize for this error.

